# Proteoglycans: Systems-Level Insight into Their Expression in Healthy and Diseased Placentas

**DOI:** 10.3390/ijms23105798

**Published:** 2022-05-21

**Authors:** Orsolya Oravecz, Andrea Balogh, Roberto Romero, Yi Xu, Kata Juhasz, Zsolt Gelencser, Zhonghui Xu, Gaurav Bhatti, Roger Pique-Regi, Balint Peterfia, Petronella Hupuczi, Ilona Kovalszky, Padma Murthi, Adi L. Tarca, Zoltan Papp, Janos Matko, Nandor Gabor Than

**Affiliations:** 1 Systems Biology of Reproduction Research Group, Institute of Enzymology, Research Centre for Natural Sciences, H-1117 Budapest, Hungary; oravecz.orsolya@ttk.hu (O.O.); balogh.andrea@ttk.hu (A.B.); ho12ember@gmail.com (K.J.); gelzsolt@gmail.com (Zs.G.); peterfiab@gmail.com (B.P.); matkojani52@gmail.com (J.M.); 2 Doctoral School of Biology, Institute of Biology, ELTE Eötvös Loránd University, H-1117 Budapest, Hungary; 3 Perinatology Research Branch, Division of Obstetrics and Maternal-Fetal Medicine, Division of Intramural Research, *Eunice Kennedy Shriver* National Institute of Child Health and Human Development, National Institutes of Health, U.S. Department of Health and Human Services (NICHD/NIH/DHHS), Bethesda, MD 20892, and Detroit, MI 48201, USA; prbchiefstaff@med.wayne.edu (R.R.); yxu@med.wayne.edu (Y.X.); rezxu@channing.harvard.edu (Z.X.); gbhatti@med.wayne.edu (G.B.); rpique@wayne.edu (R.P.-R.); atarca@med.wayne.edu (A.L.T.); 4 Department of Obstetrics and Gynecology, University of Michigan, Ann Arbor, MI 48109, USA; 5 Department of Epidemiology and Biostatistics, Michigan State University, East Lansing, MI 48824, USA; 6 Center for Molecular Medicine and Genetics, Wayne State University, Detroit, MI 48201, USA; 7 Detroit Medical Center, Detroit, MI 48201, USA; 8 Department of Obstetrics and Gynecology, Wayne State University, Detroit, MI 48201, USA; 9 Maternity Private Clinic, H-1126 Budapest, Hungary; hupuczi.petronella@maternity.hu (P.H.); pzorvosihetilap@maternity.hu (Z.P.); 10 First Department of Pathology and Experimental Cancer Research, Semmelweis University, H-1085 Budapest, Hungary; koval@korb1.sote.hu; 11 Department of Pharmacology, Monash Biomedicine Discovery Institute, Clayton, VIC 3800, Australia; padma.murthi@monash.edu; 12 Department of Obstetrics and Gynaecology, University of Melbourne, Royal Women’s Hospital, Parkville, VIC 3502, Australia; 13 Department of Computer Science, Wayne State University College of Engineering, Detroit, MI 48202, USA

**Keywords:** fetal growth restriction, placenta development, pre-eclampsia, pregnancy, proteoglycans, trophoblast differentiation, trophoblast invasion, syndecans

## Abstract

Proteoglycan macromolecules play key roles in several physiological processes (e.g., adhesion, proliferation, migration, invasion, angiogenesis, and apoptosis), all of which are important for placentation and healthy pregnancy. However, their precise roles in human reproduction have not been clarified. To fill this gap, herein, we provide an overview of the proteoglycans’ expression and role in the placenta, in trophoblast development, and in pregnancy complications (pre-eclampsia, fetal growth restriction), highlighting one of the most important members of this family, syndecan-1 (*SDC1*). Microarray data analysis showed that of 34 placentally expressed proteoglycans, *SDC1* production is markedly the highest in the placenta and that *SDC1* is the most upregulated gene during trophoblast differentiation into the syncytiotrophoblast. Furthermore, placental transcriptomic data identified dysregulated proteoglycan genes in pre-eclampsia and in fetal growth restriction, including *SDC1*, which is supported by the lower concentration of syndecan-1 in maternal blood in these syndromes. Overall, our clinical and in vitro studies, data analyses, and literature search pointed out that proteoglycans, as important components of the placenta, may regulate various stages of placental development and participate in the maintenance of a healthy pregnancy. Moreover, syndecan-1 may serve as a useful marker of syncytialization and a prognostic marker of adverse pregnancy outcomes. Further studies are warranted to explore the role of proteoglycans in healthy and complicated pregnancies, which may help in diagnostic or therapeutic developments.

## 1. Introduction

Proteoglycans are macromolecules consisting of a protein core to which glycosaminoglycan (GAG) side chains are covalently attached [1]. Most proteoglycans are important components of the extracellular matrix; however, some of them are localized to the cell surface, and serglycin is one with intracellular localization [2]. Proteoglycans regulate various cellular functions (e.g., adhesion, proliferation, migration, invasion, angiogenesis, and apoptosis) in several ways, such as by binding cytokines, growth factors, or other components of the extracellular matrix or by modulating the activation of signaling receptors mostly via interaction with GAG chains [3,4,5,6,7,8,9,10,11].

Emerging data at the RNA and protein levels have shown that more than a dozen of the nearly 50 proteoglycan core proteins [1,12] are expressed by the human placenta (Table S1). They are produced by trophoblasts (syndecans, glypican-3) and other placental cell types or decidualized stromal cells (a cell surface chondroitin-sulfate proteoglycan (CD44) and decorin) [6,13]. Placental proteoglycans are reported to be involved in different physiological functions of the placenta, such as maintenance of blood flow by anticoagulation, regulation of trophoblast migration and proliferation, and angiogenic processes as well as the regulation of inflammation [14,15,16,17,18]. Each of these functions is necessary for proper placental development and the maintenance of pregnancy. Indeed, altered placental expression of proteoglycans (e.g., syndecan-1; endocan, glypican-3) was reported in obstetrical syndromes, such as pre-eclampsia [19,20,21], fetal growth restriction [22,23,24], and gestational diabetes mellitus [25,26], pointing to their potential roles in the development of these syndromes. Furthermore, proteoglycan-attached GAGs are critical mediators of differentiation, migration, tissue morphogenesis, and organogenesis during embryonic development since embryos lacking specific GAG-modifying enzymes have distinct developmental defects [27].

Of note, the extremely rapid development of the placenta highly resembles the formation of malignant tumors. Indeed, among the properties shared by placental trophoblast cells and cancer cells are the capabilities to invade healthy tissues, form new vessels, and promote an environment that is protected from the immune system [28]. Since proteoglycans are strongly involved in various processes during tumorigenesis, which was reviewed by several workgroups [29,30,31,32,33,34,35,36,37,38,39,40,41,42,43,44,45,46,47,48], they may have similar key roles in these processes during placentation.

However, only a few studies attempted to determine the proteoglycans’ diverse roles in the placenta, and a systematic effort to provide a global view on placental proteoglycans has not been carried out. Therefore, herein we performed a systems-level analysis on proteoglycan expression in the placenta in normal and complicated pregnancies, focusing on syndecan-1 as one of the most important members of the proteoglycan family in the placenta.

## 2. Results and Discussion

### 2.1. Expression of Proteoglycans by the Placenta

**Figure 1:**
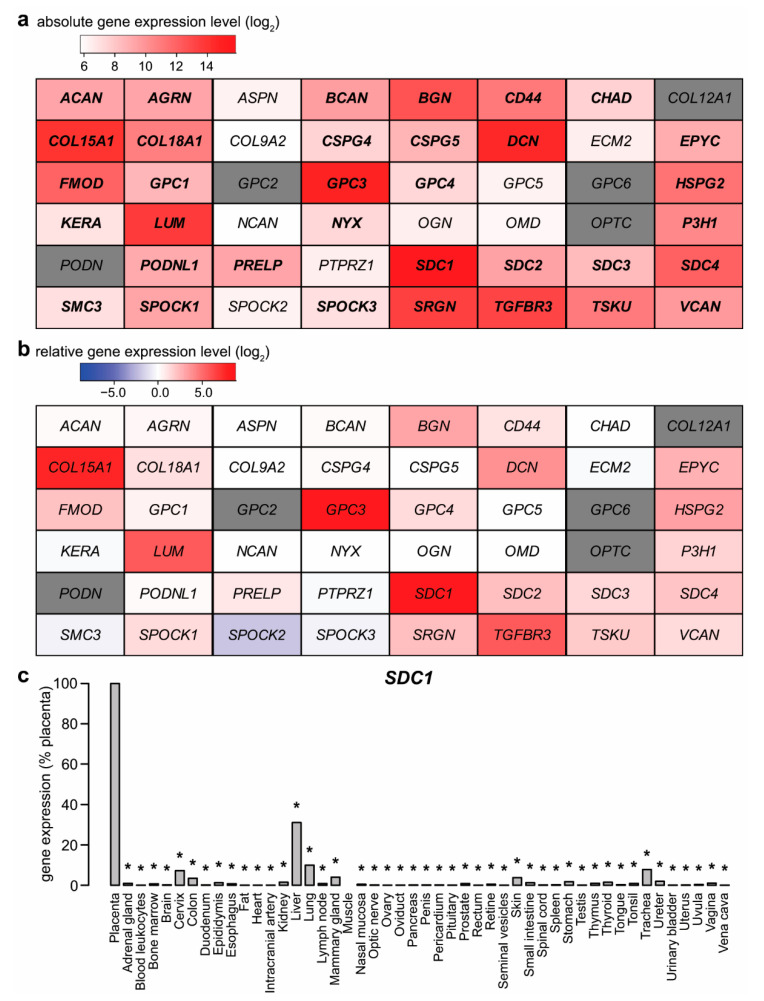
Placental expression of proteoglycan genes. (**a**) Absolute microarray gene expression values in the BioGPS database [49,50] were visualized on a heatmap. Color code depicts log_2_ gene expression levels. Bold letter: expression above threshold 100. Grey color: no data were available. (**b**) Placental expression of proteoglycan genes was compared to the median expression of 78 other tissues or cells. Differential expression of proteoglycan genes in the placenta was visualized on a heatmap. Color code depicts log_2_ gene expression ratios. Grey color: no data were available. (**c**) Real-time quantitative reverse transcription PCR (qRT-PCR) validation of BioGPS data on *SDC1* expression in human tissues (n = 3) was visualized on the diagram as the percentage of placental expression. *SDC1* mRNA expression values were normalized to *RPLP0*. Unpaired *t*-test was used for the analysis of *SDC1* expression profiling with the Human Major Tissue qPCR Array. (* *p* < 0.05). Ribosomal Protein Lateral Stalk Subunit P0—*RPLP0*; syndecan-1—*SDC1*.

To screen for proteoglycan genes expressed by the term placenta, BioGPS microarray data [49,50] were analyzed by two approaches. First, utilizing a threshold of 100 units of microarray fluorescence, 34 of the 48 proteoglycan genes were found to be expressed by the placenta (Figure 1a). The top four genes with the highest signal intensity were syndecan-1 (*SDC1*), followed by glypican-3 (*GPC3*)—both found on the surface of the parenchymal cells—decorin (*DCN*, an extracellular matrix component), and collagen, type XV, alpha 1 (*COL15A1*, a basal membrane component).

As a second approach, the placental expression for each gene was transformed as a ratio relative to the median expression in other tissues (Figure 1b). Nine proteoglycan genes had at least six times higher expression in the placenta than the median of other tissues. The top three genes—*GPC3*, *SDC1,* and *COL15A1*—had relative expression orders of magnitude: 441-fold, 390-fold, and 198-fold higher expression in the placenta than the median of other tissues, respectively. Among these genes, *GPC3* and *SDC1* also met an additional criterion, being predominantly expressed in the placenta, previously defined in papers by Than et al. and Szilagyi et al. [51,52]. Of note, a recent plasma proteome study reported the highest (26-fold) increase in the concentrations of glypican-3, amongst proteins with significant change, in the maternal circulation with advancing gestation [53].

To validate BioGPS microarray data, syndecan-1 expression results were quantified by qRT-PCR, using a TaqMan Assay and a human 48-tissue cDNA panel (Figure 1c). *SDC1* mRNA expression patterns from the qRT-PCR data were consistent with the microarray data. The highest expression was observed in the placenta, representing an approximately three-fold increase over the second-highest expression in the liver, while it has low expression in other tissues. These data suggest that proteoglycans, including *SDC1*, are important structural and molecular components of the placenta.

### 2.2. Expression Changes of Proteoglycans during Syncytiotrophoblast Differentiation

**Figure 2:**
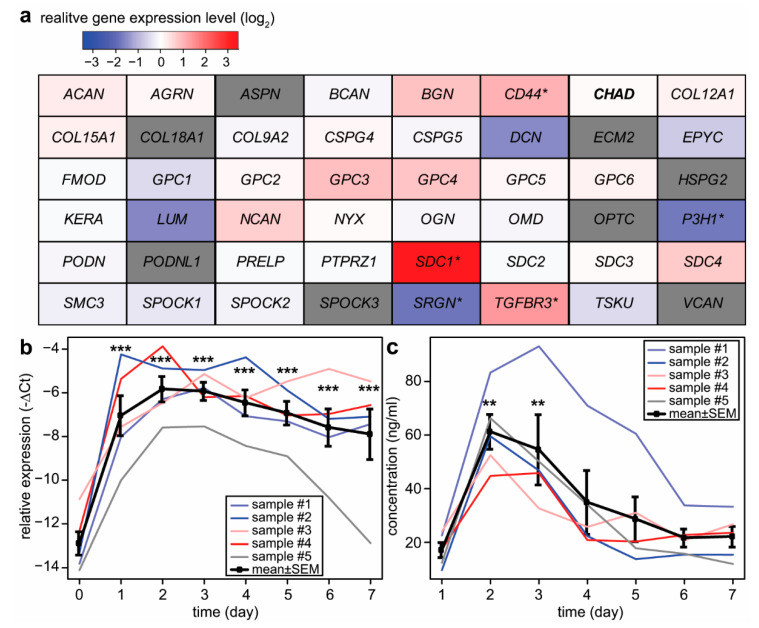
Changes in proteoglycan gene expression during villous trophoblast differentiation. (**a**) Microarray data were obtained from primary villous trophoblast cells isolated from third-trimester normal placentas (n = 3) during a seven-day differentiation period. The largest differences in gene expression compared to day 0 were visualized on a heatmap. Color code depicts log_2_ gene expression ratios. Grey color: no data were available. The original study was published by Szilagyi et al. [52]. (**b**) *SDC1* expression was monitored during villous trophoblast differentiation. qRT-PCR data were obtained from an extended set of primary villous trophoblast cells isolated from third-trimester normal placentas (n = 5) during a seven-day differentiation period. Relative expression of *SDC1*, normalized to *RPLP0*, was visualized on the diagraph. (**c**) Changes in syndecan-1 protein concentration in cell culture supernatants (n = 5) were examined throughout spontaneous syncytial differentiation of primary villous trophoblast cells. One-Way ANOVA with Dunnett’s post-hoc test was used for the analysis of qRT-PCR and ELISA results (* *p* < 0.05, ** *p* < 0.01, *** *p* < 0.001). Ribosomal Protein Lateral Stalk Subunit P0—*RPLP0*; syndecan-1—*SDC1*.

Since one of the most important processes in placental development is the differentiation of cytotrophoblast cells into the syncytiotrophoblast, change in the expression of proteoglycan genes was investigated in an in vitro trophoblast differentiation model system. The basic experiment involved the spontaneous syncytial differentiation of isolated primary cytotrophoblast cells [52]. The gene expression pattern of these cells was measured daily by microarray for seven days. The maximal difference in the expression of proteoglycan genes compared to the baseline day 0 was ascertained. *SDC1* showed the highest difference in the course of syncytial trophoblast differentiation with a 10.9-fold increase in expression on day 2 (Figure 2a). Of note, *CD44* and transforming growth factor-beta receptor III (*TGFBR3*) were also upregulated with a 2.1-fold and 2.6-fold increase, respectively, while leprecan-1—also known as prolyl 3-hydroxylase 1 (*P3H1*)—(3.4-fold) and serglycin (*SRGN*) (−3.5-fold)—was downregulated. To validate the microarray results, the expression of *SDC1* mRNA was measured by qRT-PCR in cell lysates, and the protein level was monitored in cell culture supernatants by ELISA. The maximum mRNA expression of syndecan-1 was detected in the cells on day 2 after seeding with an approximately 120-fold increase in expression (Figure 2b). The protein expression in the supernatant, correspondingly, showed the same time dependence (Figure 2c).

**Figure 3:**
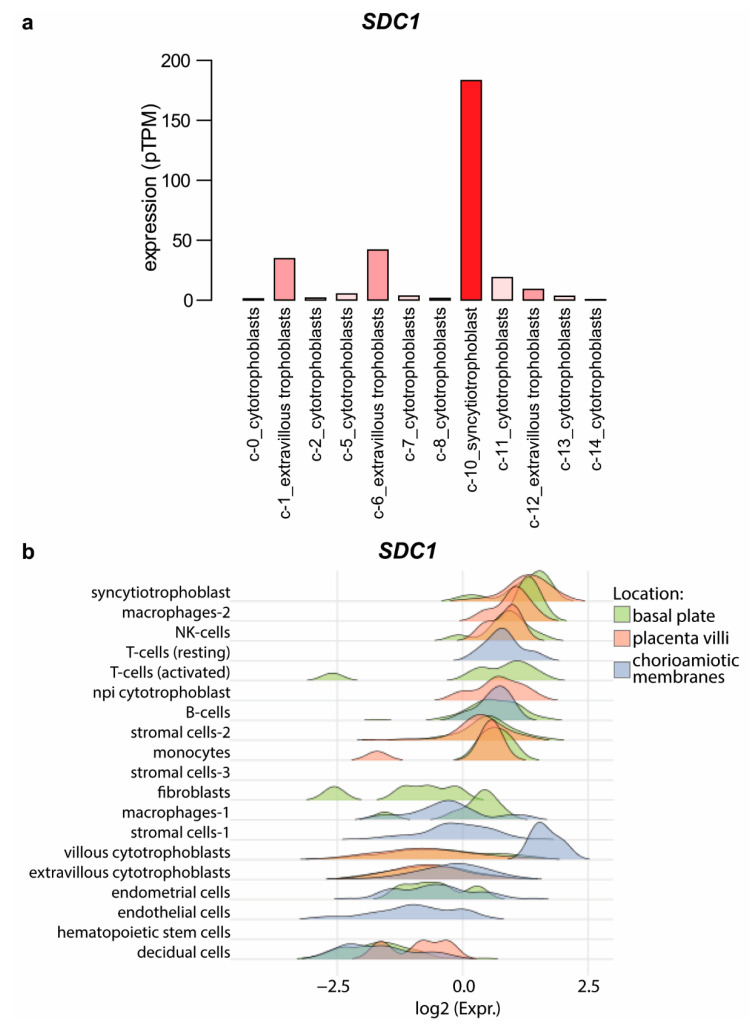
*SDC1* expression in the placenta. (**a**) Single-cell RNA-seq data, derived from first-trimester placentas, were downloaded from the Human Protein Atlas [56]. The original study was performed by Vento-Tormo et al. [55]. *SDC1* mRNA expression (pTPM) in trophoblast cell clusters (colored with shades of red) was visualized on a bar chart. (**b**) Single-cell RNA-seq data, derived from term placentas, were downloaded from http://genome.grid.wayne.edu/sclabor/ (accessed on 5 April 2022). The study was performed by Pique-Regi et al. [57]. *SDC1* mRNA expression (normalized log_2_ count data) in cell clusters in three placental compartments (basal plate, placenta villi, and chorioamniotic membranes) was visualized on a histogram chart. Non-proliferative interstitial—npi; protein-transcripts per million—pTPM.

Cuffdiff analysis of RNA-seq data of Azar et al. found that *DCN*, lumican (*LUM*), and *P3H1* were downregulated in the syncytiotrophoblast, while edgeR analysis of the same dataset revealed the downregulation of collagen type XII alpha 1 chain (*COL12A1*), *P3H1*, syndecan-4 (*SDC4*), and structural maintenance of chromosomes 3 (*SMC3*) as well as the upregulation of *GPC3* [54]. In addition, single-cell RNA-seq data derived from the Human Protein Atlas also supports that the syncytiotrophoblast (isolated from first-trimester placentas) has the highest *SDC1* expression compared to other trophoblast types in the placenta (Figure 3a) [55,56]. At term, expression of *SDC1* remains the highest in the syncytiotrophoblast, as evidenced by a recent single-cell RNA-seq study searching for single-cell transcriptional signatures of the human placenta in term and preterm parturition (Figure 3b) [57].

At the protein level, syndecan-1 was also primarily detected in the syncytiotrophoblast, a multinucleated cell layer of the human placenta. In the syncytiotrophoblast, syndecan-1 was localized to the cytoplasm and the apical cell surface. Immunohistochemical examinations showed negative staining for other cell types of the placenta, e.g., villous trophoblasts and stromal cells of chorionic villi [4,21]. In a recent publication revealing the pivotal role of the transcriptional co-activator yes-associated protein in trophoblast stemness of the developing human placenta, syndecan-1 was even used as a cell fusion marker [58]. Furthermore, during a healthy pregnancy, serum concentrations of syndecan-1 increase steadily with gestational age, in line with the growing placenta and syncytiotrophoblast volume [59,60,61].

### 2.3. Expression Changes of Proteoglycans during Extravillous Trophoblast Differentiation

**Figure 4:**
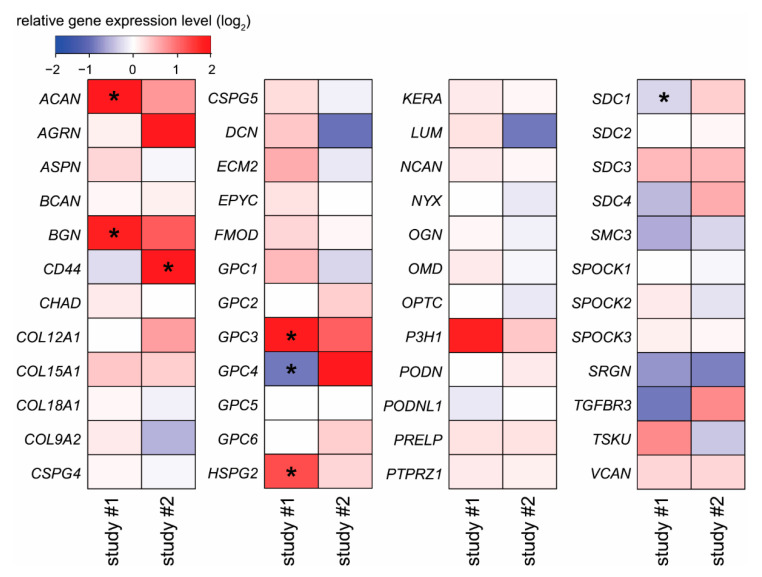
Differentially expressed proteoglycan genes in invasive extravillous trophoblast cells compared to villous cytotrophoblast cells. Microarray data from two independent GEO (study #1) [63] or ArrayExpress (study #2) [13] datasets were reanalyzed. Color code depicts log_2_ gene expression ratios. Red indicates higher gene expression in extravillous trophoblasts, while blue depicts higher gene expression in villous cytotrophoblasts. Stars show significant differences.

Cytotrophoblast stem cells also differentiate into extravillous trophoblasts [62], which facilitate critical tissue connection in the developing placental-uterine interface. Therefore, two independent datasets from Gene Expression Omnibus (GEO) and ArrayExpress were used for the analysis to identify proteoglycan expression patterns associated with extravillous trophoblast differentiation. In both studies, mRNA signatures of purified villous trophoblasts (poor invasiveness) and extravillous trophoblasts (high invasiveness), isolated from first-trimester human placentas, were compared by using GeneChip analyses [13,63]. We focused on those genes whose expression was found to be altered in the same direction in both datasets and which attained statistical significance at least in one dataset. Four genes, aggrecan (*ACAN*), biglycan (*BGN*), *GPC3*, and heparan sulfate proteoglycan 2 (*HSPG2*), showed higher expression in extravillous trophoblasts in both datasets (Figure 4).

**Figure 5:**
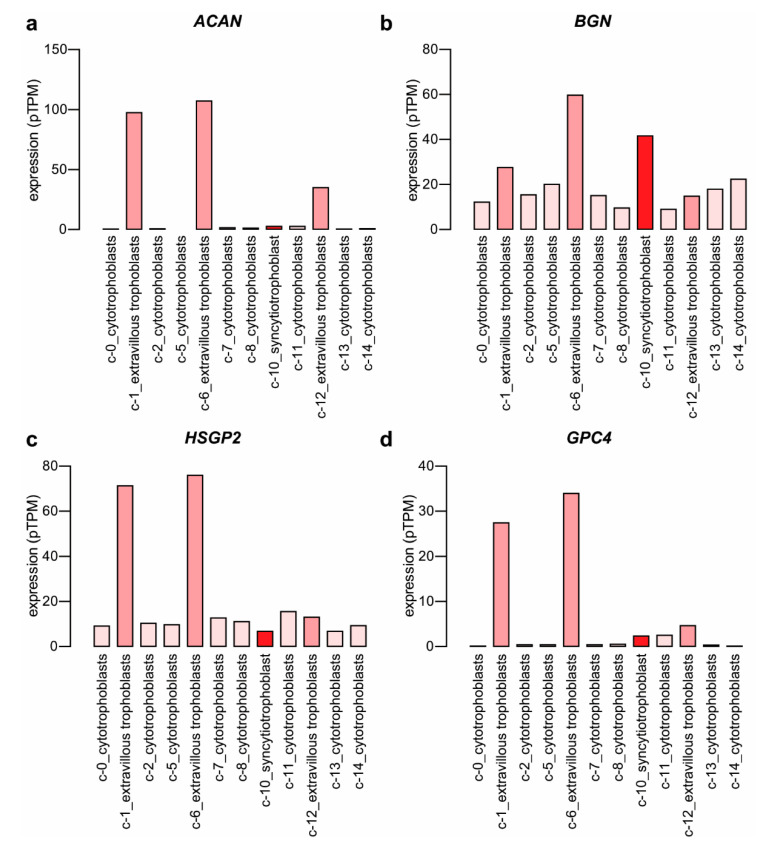
Expression of selected proteoglycan genes in the placenta. Single-cell RNA-seq data were downloaded from the Human Protein Atlas [56]. The original study was performed by Vento-Tormo et al. [55]. *ACAN* (**a**), *BGN* (**b**), *HSPG2* (**c**), and *GPC4* (**d**) mRNA expression (pTPM) in trophoblast cell clusters (colored with shades of red) were visualized on a bar chart. Protein-transcripts per million—pTPM.

Upregulated expression of *ACAN*, *BGN*, and *HSPG2* was also supported by single-cell RNA-seq data relative to other trophoblast cell clusters (Figure 5a–c) retrieved from the Human Protein Atlas [56]. Single-cell RNA-seq data of glypican-4 (*GPC4*) (Figure 5d) and *SDC1* (Figure 3) expression rather supports the microarray study of Apps et al. [13].

### 2.4. Placental Expression Changes of Proteoglycans in Pre-Eclampsia and Fetal Growth Restriction

**Figure 6:**
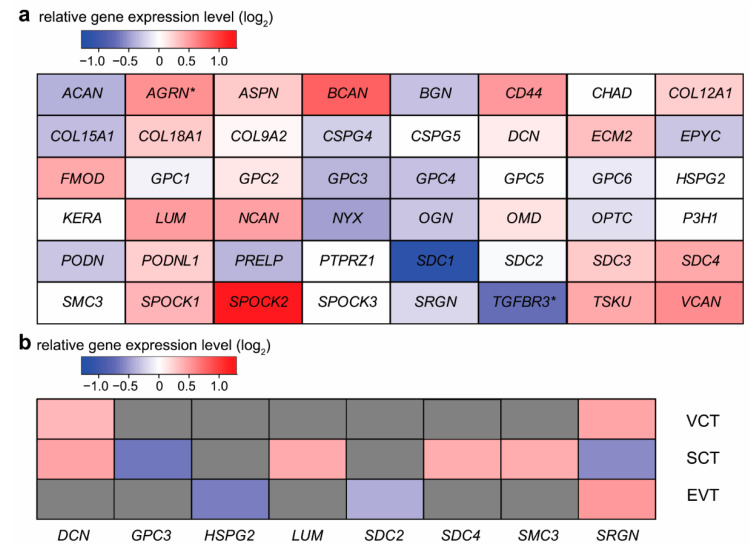
Placental proteoglycan gene expression in pre-eclampsia. (**a**) Placental microarray data from women with early-onset pre-eclampsia (n = 12) and preterm controls (n = 5) were published by Than et al. [51]. Color code depicts log_2_ gene expression ratios between cases and controls. Red indicates higher gene expression, while blue depicts lower gene expression in pre-eclampsia. Stars show significant differences. (**b**) Single-cell RNA-seq data from women with early-onset pre-eclampsia and preterm controls were published by Zhang et al. [70]. Differentially expressed genes in villous cytotrophoblasts (VCT), in the syncytiotrophoblast (SCT), and in extravillous trophoblasts (EVT) were visualized on a heatmap. Red indicates higher gene expression, blue depicts lower gene expression in pre-eclampsia (log_2_ fold change, q-value < 0.05), and grey depicts non-differentially expressed genes.

As proteoglycans have important anticoagulant properties in vascular endothelial environments, including the uteroplacental circulation, we and others have examined their placental expression in pregnancies complicated by pre-eclampsia [20] or fetal growth restriction [22,23,24], the two clinical entities having a common etiological background, including abnormal uteroplacental circulation and an antiangiogenic state [64,65,66,67,68,69]. In general, only slight alterations in the mRNA expression of different proteoglycan genes were detectable in placental samples derived from women with early-onset pre-eclampsia compared to gestational-age-matched controls, in the study of Than et al. [51] (Figure 6a).

The greatest changes were detected for sparc/osteonectin, cwcv, and kazal-like domains proteoglycan 2 (*SPOCK2*), *SDC1*, brevican (*BCAN*), transforming growth factor-beta receptor III (*TGFBR3*), versican (*VCAN*), and agrin *(AGRN*) with 2.4-, −2.2-, 1.7-, −1.6-, 1.5-, and 1.5-fold changes, respectively (Figure 6a). However, only *AGRN* and *TGFBR3* had a significant change in expression in early-onset pre-eclampsia.

A comprehensive analysis of high-quality RNA-seq data also found *TGFBR3* as the most downregulated gene in pre-eclampsia, while *SPOCK2*, glypican-5 (*GPC5*)*,* and chondroitin sulfate proteoglycan 4 (*CSPG4*) were significantly upregulated [71]. In addition, expression changes in the placenta, obtained from women with preterm pre-eclampsia and from healthy controls, were also revealed in a small single-cell RNA-seq study [70]. *DCN* and *SRGN* were upregulated in villous cytotrophoblasts; *GPC3* and *SRGN* were downregulated, while *DCN*, *LUM, SDC4*, and *SMC3* were upregulated in the syncytiotrophoblast; *HSPG2* and syndecan-2 (*SDC2*) were downregulated, and *SRGN* was upregulated in extravillous trophoblasts (Figure 6b). When comparing the results of these studies, it transpires that gene expression patterns were similar in the bulk placenta to that of the syncytiotrophoblast at the level of some proteoglycans, probably due to their predominant expression in that cell compartment. Herein, we did not perform a meta-analysis since different patient groups, and different basic methods limit the comparability of numerous studies. However, we investigated transcriptomic changes in pre-eclampsia in a recent publication [72].

In the case of fetal growth restriction, four microarray studies [66,73,74,75] and one RNA-seq study [76] were available. Downregulation of *SDC1* [74] and upregulation of epiphycan (*EPYC*) and sparc/osteonectin, cwcv, and kazal-like domains proteoglycan 1 (*SPOCK1*) [75] could be detected. Furthermore, Whitehead et al. measured circulating placenta-specific genes by microarray in maternal blood at 26–30 weeks’ gestation to identify pregnancies at risk of late-onset fetal growth restriction [77] and found *BGN* and *GPC3* to be upregulated in fetal growth restriction.

At the protein level, the expression of five proteoglycans (decorin, glypican-1, glypican-3, perlecan, syndecan-2) of the nine measured was significantly decreased in the placentas of women with pre-eclampsia compared to gestational-age-matched controls [20]. Regarding perlecan protein levels in the placenta, our study found the same direction of change in late-onset pre-eclampsia, which constitute the majority of cases, while an opposite directional change was observed in early-onset pre-eclampsia [78]. This may reflect the distinct placental pathologies of these clinically different pre-eclampsia subtypes.

Although reduced *SDC1* expression was not significant in microarray and single-cell RNA-seq analyses, syndecan-1 expression levels in the placenta, using immunohistochemistry and/or qRT-PCR, were shown to be reduced during pre-eclampsia, as reported by a few publications [19,60,79]. In line with reduced syndecan-1 expression in placentas with pre-eclampsia, serum levels of syndecan-1 were found to be lower in pre-eclamptic patients compared to healthy controls [21,61,80,81]. Only one study found that plasma concentration of syndecan-1 was similar in pre-eclamptic and normotensive pregnant women [82], and one showed significantly elevated serum concentrations of syndecan-1 in women with hemolysis, elevated liver enzymes, low platelet count (HELLP) syndrome [59]. Although Szabó et al. found lower maternal serum concentrations of syndecan-1 in women with pre-eclampsia than in healthy controls, their study revealed higher immunostaining intensity of the syncytiotrophoblast brush border membrane in preterm pre-eclampsia and HELLP syndrome, which may be attributable to the redistribution of syndecan-1 from the plasma toward the cell surface in these syndromes [21]. This is interesting in the light that cellular redistribution of placenta-specific glycan-binding proteins, galectins [83,84,85,86], in the syncytiotrophoblast was also observed in placentas of women with preterm pre-eclampsia [87,88,89]. It is possible that such cellular redistribution of placenta-specific glycan and glycan-binding proteins are linked together as a result of the activation of an ischemic-inflammatory disease pathway in the tropohoblast in preterm pre-eclampsia [90,91]. Interestingly, one study found lower syndecan-1 maternal plasma concentrations in pre-eclampsia (but not gestational hypertension) already at the 20th week of gestation, when clinical signs have not yet been developed [60]. This means that biological pathways leading to decreased syndecan-1 levels in the placenta and consequently in the maternal circulation are present in midgestation. Indeed, this is true for the above-mentioned ischemic inflammatory disease pathways, as we demonstrated in a previous study [51]. This also suggests that syndecan-1 measurements in maternal circulation may have predictive or prognostic value for pre-eclamptic pregnancies. However, large longitudinal studies need to investigate this phenomenon.

In the case of fetal growth restriction, the mRNA and protein expression levels of both syndecan-1 and syndecan-2 were significantly decreased in diseased placentas compared to the controls [23]. However, according to Garcha et al., syndecan-1 serum levels, but not placental protein levels, were reduced significantly in pregnancies complicated by fetal growth restriction [92]. Overall, these partly contrasting data highlight the complexity of the phenomenon, which requires further studies for a deeper understanding of the proteoglycans’ behavior in the placenta in various obstetrical syndromes, which may help diagnostic or therapeutic developments.

### 2.5. Potential Functional Significance of Syndecan-1 in the Placenta

Microarray data showed that of the proteoglycans expressed in the placenta, *SDC1* production is markedly the highest. Moreover, during villous trophoblast differentiation into the syncytiotrophoblast, *SDC1* has the highest upregulation among all proteoglycans. Furthermore, *SDC1* gene expression (microarray and RNA-seq data), although not always significantly, is found to be downregulated in pre-eclampsia and fetal growth restriction, which corroborates with a significantly lower concentration of syndecan-1 protein in the maternal blood in these obstetrical syndromes. These findings, along with data on the role of syndecan-1 in regulating cytoskeleton remodeling, cell adhesion, growth factor signaling, anchorage-dependent growth, and invasiveness in different cell systems [3,93,94], suggest that it may play important roles in the maternal-fetal interface.

On the fetal side of the placenta, the critical involvement of syndecan-1 in trophoblast syncytialization [58] has been reported by a few studies. Prakash et al. found that syndecan-1 silencing led to a decrease in cell fusion and even human chorionic gonadotropin (hCG) production in trophoblast-like BeWo choriocarcinoma cells. Their explanation was that the impairment in the establishment of cell-to-cell contact is essential for the morphological differentiation leading to cell fusion. The possible ways through which syndecans, e.g., syndecan-1, regulate the cellular function may include supporting cell adhesion via the binding of adhesion molecules such as fibronectins, collagens, and thrombospondin [94] or may help to establish feto-maternal communication through binding with growth factors [4]. In addition, syndecans have been found to serve as primary attachment receptors for a disintegrin and metalloproteinase 12 (ADAM12) in various human and mouse carcinoma and fibroblast cell lines [95], but the same stands for other cells as well. In the case of trophoblasts, ADAM12 is able to shed the ectodomain of E-cadherin and potentiate trophoblast fusion [96]. The importance of syndecan-1 has been revealed in the maintenance of intestinal barrier function [97,98]. In this context, Moore et al. found that syncytialization caused a marked change in syndecans, matching observations of placental extracellular matrices. Furthermore, the barrier function of the extracellular matrix, as measured by electric cell-substrate impedance sensing, increased significantly during and after syncytialization [98].

Interestingly, the role of syndecan-1 also emerges in trophoblast invasion in association with ADAM12. ADAM12, upregulated during extravillous trophoblast differentiation, was suggested to exert its promigratory function in extravillous trophoblasts by inducing integrin beta 1-mediated cellular spreading [99]. As syndecans are key receptors for ADAM12, they may support the spreading.

On the maternal side of the placenta, it was demonstrated by the workgroup of Hess that syndecan-1 is involved in the control of apoptosis both in human endometrial epithelial [100] and in stromal cells [101]. *SDC1* knockdown in endometrial epithelial cells resulted in the decreased expression of several anti-apoptotic proteins, a cellular inhibitor of apoptosis (cIAP)-1 and -2, X-linked inhibitor of apoptosis (XIAP), survivin, clusterin, heme oxygenase (HO-2), and heat shock proteins HSP27 and -70. Correspondingly, active caspase-3 was increased more severely in knockdown cells after treatment with implantation-related stimuli [100]. In endometrial stromal cells, active caspase-3 was also increased in knockdown cells after treatment with implantation-related stimuli [101]. Pro-apoptotic BCL2-associated agonist of cell death (Bad) and Fas receptor increased in accordance with decidualization, and *SDC1* knockdown further increased Bad expression. On the contrary, anti-apoptotic Livin decreased due to decidualization and knockdown of *SDC1*. Implantation stimuli provoked an increase of abundant proteins in all different cell types (stromal cell, *SDC1* knockdown stromal cells, decidualized stromal cells, and decidualized *SDC1* knockdown stromal cells) in varying degrees. Bad was upregulated in all four cell types, whereas anti-apoptotic cIAP-1 and XIAP were downregulated only in knockdown cells [101]. Altogether, these findings suggest that syndecan-1 may affect the fine-tuning of apoptosis in the endometrium, regulating the embryo’s invasion depth as a crucial step for regular implantation followed by successful pregnancy [100,101].

**Figure 7:**
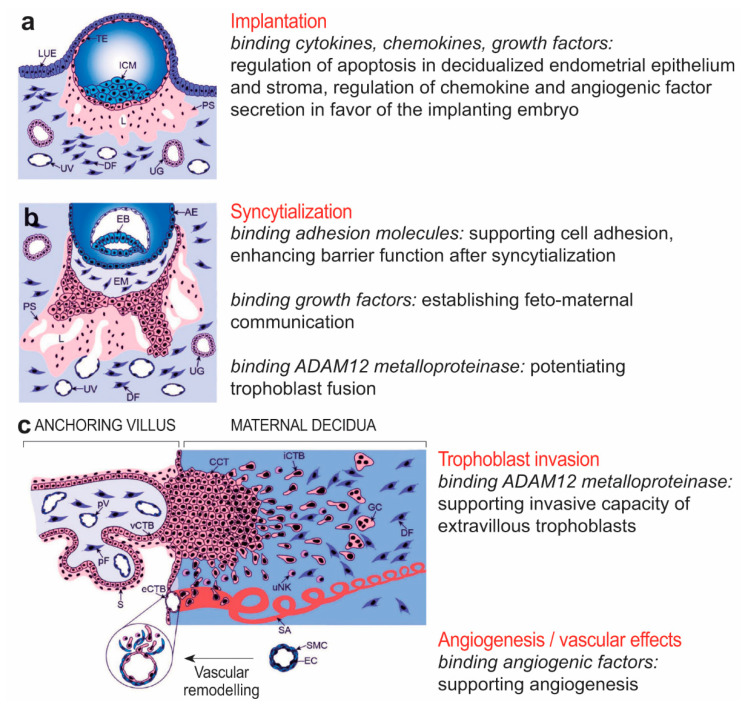
Physiological aspects of syndecan-1 at the maternal-fetal interface. The figure represents multiple roles of syndecan-1 in implantation, angiogenesis, maternal-fetal immune tolerance, and trophoblast invasion. (**a**) Embryo implantation. (**b**) Formation of primary villi by proliferative cytotrophoblasts and syncytialization. (**c**) Formation of tertiary villi, placental angiogenesis, extravillous trophoblast invasion, and spiral artery remodeling. Amniotic epithelium, AE; cell column trophoblast, CCT; dendritic cell, DC; decidual fibroblast, DF; embryoblast, EB; endothelial cell, EC; extracellular matrix, ECM; extraembryonic mesoderm, EM; endovascular cytotrophoblast, eCTB; giant cell, GC; inner cell mass, ICM; interstitial cytotrophoblast, iCTB; luminal uterine epithelium, LUE; lacunae, L; matrix metalloproteinase, MMP; natural killer, NK; placental fibroblast, pF; primitive syncytium, PS; placental vessel, pV; spiral artery, SA; syncytium, S; smooth muscle cell, SMC; trophectoderm, TE; uterine gland, UG; uterine NK cell, uNK; uterine vessel, UV; villous cytotrophoblast, vCTB. Cartoons are adapted from Knöfler and Pollheimer [103] under the terms of the Creative Commons Attribution License.

Of note, the same workgroup found that *SDC1* knockdown led to significant changes in cytokine, chemokine, growth factors, and angiogenic factor (e.g., C-X-C motif chemokine ligand 1 (CXCL1), CXCL8, C-C motif chemokine ligand 2 (CCL2), hepatocyte growth factor (HGF)) expression profiles of human endometrial stromal cells [102]. Incubation with IL-1β altered the expression patterns of chemokines and angiogenic factors toward inflammatory-associated molecules and factors involved in extracellular matrix regulation. Therefore, syndecan-1 appears to play an important role as a co-receptor and storage factor for many cytokines, chemokines, growth factors, and angiogenic molecules during the decidualization and implantation period, supporting proper implantation and angiogenesis by regulation of chemokine and angiogenic factor secretion in favor of the implanting embryo (Figure 7) [102].

Of importance, the syndecan-1 ectodomain can be shed by various matrix metalloproteinases in a highly regulated fashion, and soluble syndecan-1 can compete with the intact one for extracellular ligands. The multiple roles of syndecan shedding, including inflammation, wound healing, and tumor progression, were reviewed by Manon-Jensen et al. [104]. It would be important to reveal the regulation of syndecan shedding and functions of shed syndecans in pregnancy as well.

## 3. Summary and Conclusions

In this review, we aimed to characterize the expression and potential functions of all proteoglycans at the maternal-fetal interface in healthy and diseased pregnancies. Importantly, we detected *SDC1* to be primarily expressed by the placenta among all human tissues and primarily in the syncytiotrophoblast within the placenta, where it is localized to the cytoplasm and the apical cell surface, the largest interface between the maternal and fetal compartments. Microarray data showed that of 34 proteoglycans expressed in the placenta, *SDC1* production is markedly the highest. Moreover, during villous trophoblast differentiation into the syncytiotrophoblast, *SDC1* has the highest upregulation among all proteoglycans. We also analyzed transcriptomic data of the placenta from various obstetrical syndromes and identified *SDC1*, among other dysregulated proteoglycan genes, in pre-eclampsia and fetal growth restriction. In line with these findings, syndecan-1 concentrations are decreased in the maternal blood in these syndromes, where placental development and malfunction are at the center of the disease. Since syndecan-1 supports proper implantation and angiogenesis by the regulation of chemokine and angiogenic factor secretion in favor of the implanting embryo, it may have a key role in the development of placental dysfunction in obstetrical syndromes, where these functions are severely affected.

In conclusion, proteoglycans are important components of the placenta and regulate various steps of placental development as well as participate in the maintenance of healthy pregnancy. Syndecan-1, with the highest expression among proteoglycans, may serve as a useful marker of syncytialization and may also be a diagnostic/prognostic marker of adverse pregnancy outcomes, such as pre-eclampsia and fetal growth restriction. Further studies are warranted to explore the role of proteoglycans, including syndecan-1, in healthy pregnancies and in pregnancies with adverse outcomes to better characterize molecular signaling pathways and to reveal potential therapeutic targets.

## 4. Materials and Methods

### 4.1. Placental Expression of Proteoglycan Coding Genes

We downloaded the human U133A/GNF1H microarray data on 79 human tissues from the BioGPS database [105]. In the case of absolute mRNA expression, the threshold was set to 100. To demonstrate gene expression in the placenta on a relative scale, in comparison to other kinds of tissues, the relative placental expression was calculated relative to the median Q50 value of all other tissues.

Microarray data of trophoblast differentiation into the syncytiotrophoblast were downloaded from GEO (Acc. No.: GSE130339) [52]. Microarray data of extravillous trophoblast differentiation were downloaded from GEO (Acc. No.: GSE9773) [63] and the ArrayExpress microarray data repository (Acc. No.: E-MTAB-429) [13]. Microarray data of placentas obtained from women with preterm pre-eclampsia with or without HELLP syndrome (n = 12), as well as gestational-age-matched controls (n = 5), were downloaded from GEO (Acc. No.: GSE66273) [51,106].

Single-cell RNA-seq data (pTPM) were downloaded from the Human Protein Atlas [107]. The 19 cell clusters were narrowed down to the 12 trophoblast clusters, excluding fibroblasts, endothelial cells, and Hofbauer cells. The original study was executed by Vento-Tormo et al. [55].

### 4.2. Isolation of Primary Trophoblasts and Cell Culturing

Placentas (n = 6) were collected prospectively from normal pregnant women at term, delivered by cesarean section, at Hutzel Women’s Hospital of the Detroit Medical Center (Detroit, MI, USA), and were then processed at the Perinatology Research Branch, NICHD/NIH/DHHS, for in vitro trophoblast experiments. For cytotrophoblast isolation, we used the modified method of Kliman et al. [108]. One hundred grams of villous trophoblast were cut from the placenta, then washed with PBS. The placenta pieces were sequentially digested with 60 U/mL Dnase I (Sigma-Aldrich, St. Louis, MI, USA) and 0.25% (Thermo Fisher Scientific, Waltham, MA, USA) trypsin for 90 min at 37 °C, then the digested cells were filtered through 100 μm Falcon nylon mesh cell strainers (BD Biosciences, San Jose, CA, USA) and the erythrocytes were lysed using 5 mL NH_4_Cl solution (Stemcell Technologies, Vancouver, BC, Canada). The cells were then washed and resuspended, layered over 20–50% Percoll gradients, and centrifuged at 1200× *g* for 20 min. Negative selection was used to collect trophoblast-containing bands and to exclude non-trophoblastic cells, using anti-CD14 (20 μg/mL) and anti-CD9 (20 μg/mL) mouse monoclonal antibodies (R&D Systems, Minneapolis, MN, USA), MACS anti-mouse IgG microbeads, and MS columns (Miltenyi Biotec, Auburn, CA, USA). Next, the primary trophoblast cells were plated on a collagen-coated 12-well plate (BD Biosciences) in Iscove’s modified Dulbecco’s medium (IMDM, Thermo Fisher Scientific) and completed with 1% penicillin/streptomycin and 10% fetal bovine serum (FBS). Primary trophoblasts were kept in IMDM containing 1% penicillin/streptomycin and 5% non-pregnant human serum (SeraCare, Milford, MA, USA) to test the effect of trophoblast differentiation on selected genes’ expression. Every 24 h, the medium was replenished, and all 24 h cells were harvested for total RNA between days 1 and 7.

### 4.3. Total RNA Isolation, cDNA Generation, and RT-qPCR

Total RNA was isolated from cells in 12-well plates each day with RNeasy Mini Kit and RNase-Free DNase Set (Qiagen, Valencia, CA, USA). RNA concentrations were measured with a NanoDrop1000 Spectrophotometer (Thermo Fisher Scientific). Total RNA (500 ng) was reverse-transcribed with SuperScript III First-Strand Synthesis Kit (Invitrogen-Thermo Fisher Scientific). TaqMan Assays (Applied Biosystems-Thermo Fisher Scientific) for *SDC1* (Hs00896423_m1) and *RPLP0* (large ribosomal protein; Endogenous Control; 4326314E) were used for gene expression profiling on an ABI 7500 Fast Real-Time PCR System (Applied Biosystems-Thermo Fisher Scientific). All experiments were run in triplicate.

### 4.4. Human 48-Tissue cDNA Panel Expression Profiling

The same TaqMan Assays for *SDC1* and *RPLP0* were also used for gene expression profiling on Human Major Tissue qPCR Arrays containing first-strand cDNAs from 48 different pooled tissues (n = 3), OriGene Technologies, Inc., Rockville, MD, USA) on an ABI 7500 Fast Real-Time PCR System (Applied Biosystems-Thermo Fisher Scientific). All experiments were run in triplicate.

### 4.5. Syndecan-1 Immunoassay

Syndecan-1 concentrations in supernatants of primary trophoblast cell cultures (n = 5) were measured with a human syndecan-1 sandwich ELISA Kit (Cell Sciences, Canton, MA, USA), according to the manufacturer’s instructions. The sensitivity of the assay was <2.56 ng/mL, and the coefficients of intra-assay variation and inter-assay variation were 7.6% and 6.8%, respectively. All experiments were run in triplicate.

### 4.6. Statistical Analysis

Statistical analysis was performed using GraphPad Prism 5.0 (GraphPad Software, San Diego, CA, USA). Unpaired *t*-test was used for the analysis of *SDC1* expression profiling with the Human Major Tissue qPCR Array. One-Way ANOVA test with Dunnett’s post-hoc test was used for the analysis of qRT-PCR and ELISA results. Results were considered statistically significant at * *p* < 0.05, ** *p* < 0.01, and *** *p* < 0.001.

## Data Availability

Not applicable.

## Supplementary Materials

The following supporting information can be downloaded at: https://www.mdpi.com/article/10.3390/ijms23105798/s1.
