# Supplementary material for: Proteoglycans: Systems-Level Insight into Their Expression in Healthy and Diseased Placentas
Source: Int J Mol Sci. 2022 May 21;23(10):5798. doi: 10.3390/ijms23105798 (PMC9147780; doi:10.3390/ijms23105798)
Supplement: Supplementary file 1 [file ijms-23-05798-s001.zip › ijms-1722203-supplementary.pdf]

Supplementary material

Supplementary Table S1. List of proteoglycan genes together with those reported to be expressed by the placenta.

Entrez Gene ID	Gene Symbol	Gene Name	Reported in placenta
176	*ACAN*	aggrecan	
375790	*AGRN*	agrin	[1]
54829	*ASPN*	asporin	
63827	*BCAN*	brevican	
633	*BGN*	biglycan	[2-4]
960	*CD44*	CD44 molecule (Indian blood group)	[5-8]
1101	*CHAD*	chondroadherin	
1303	*COL12A1*	collagen, type XII, alpha 1	[9]
1306	*COL15A1*	collagen, type XV, alpha 1	[10]
80781	*COL18A1*	collagen, type XVIII	[11]
1298	*COL9A2*	collagen, type IX, alpha 2	
1464	*CSPG4*	chondroitin sulfate proteoglycan 4	[12]
10675	*CSPG5*	chondroitin sulfate proteoglycan 5 (neuroglycan C)	
1634	*DCN*	decorin	[13-17]
1842	*ECM2*	extracellular matrix protein 2, female organ and adipocyte specific	
1833	*EPYC*	epiphycan	
2331	*FMOD*	fibromodulin	
2817	*GPC1*	glypican-1	[18-20]
221914	*GPC2*	glypican-2	
2719	*GPC3*	glypican-3	[20-22]
2239	*GPC4*	glypican-4	
2262	*GPC5*	glypican-5	
10082	*GPC6*	glypican-6	
3339	*HSPG2*	heparan sulfate proteoglycan 2 (perlecan)	[23]
11081	*KERA*	keratocan	
64175	*P3H1* *(LEPRE1)*	prolyl 3-hydroxylase 1 (leucine proline-enriched proteoglycan, leprecan-1)	
4060	*LUM*	lumican	[24, 25]
1463	*NCAN*	neurocan	
60506	*NYX*	nyctalopin	
4969	*OGN*	osteoglycin	
4958	*OMD*	osteomodulin	
26254	*OPTC*	opticin	
127435	*PODN*	podocan	[26]
79883	*PODNL1*	podocan-like 1	
5549	*PRELP*	proline/arginine-rich end leucine-rich repeat protein	
5803	*PTPRZ1*	protein tyrosine phosphatase, receptor-type, Z polypeptide 1	
6382	*SDC1*	syndecan-1	[27-29]
6383	*SDC2*	syndecan-2	[30]
9672	*SDC3*	syndecan-3	[30]
6385	*SDC4*	syndecan-4	[18, 30, 31]
9126	*SMC3*	structural maintenance of chromosomes 3 (bamacan)	
6695	*SPOCK1*	sparc/osteonectin, cwcv and kazal-like domains proteoglycan (testican-1)	

9806	*SPOCK2*	sparc/osteonectin, cwcv and kazal-like domains proteoglycan (testican 2)	
50859	*SPOCK3*	sparc/osteonectin, cwcv and kazal-like domains proteoglycan (testican 3)	
5552	*SRGN*	serglycin	
7049	*TGFBR3*	transforming growth factor, beta receptor III (betaglycan)	[32-34]
25987	*TSKU*	tsukushi, small leucine rich proteoglycan	
1462	*VCAN*	versican	

References

1. Chen, C. P.; Liu, S. H.; Lee, M. Y.; Chen, Y. Y., Heparan sulfate proteoglycans in the basement membranes of the human placenta and decidua. *Placenta* **2008,** 29, (4), 309-16.
2. Wiberg, C.; Hedbom, E.; Khairullina, A.; Lamande, S. R.; Oldberg, A.; Timpl, R.; Morgelin, M.; Heinegard, D., Biglycan and decorin bind close to the n-terminal region of the collagen VI triple helix. *The Journal of biological chemistry* **2001,** 276, (22), 18947-52.
3. Goryszewska-Szczurek, E.; Baryla, M.; Kaczynski, P.; Waclawik, A., Prokineticin 1-prokineticin receptor 1 signaling in trophoblast promotes embryo implantation and placenta development. *Sci Rep* **2021,** 11, (1), 13715.
4. Chui, A.; Gunatillake, T.; Brennecke, S. P.; Ignjatovic, V.; Monagle, P. T.; Whitelock, J. M.; van Zanten, D. E.; Eijsink, J.; Wang, Y.; Deane, J.; Borg, A. J.; Stevenson, J.; Erwich, J. J.; Said, J. M.; Murthi, P., Expression of Biglycan in First Trimester Chorionic Villous Sampling Placental Samples and Altered Function in Telomerase-Immortalized Microvascular Endothelial Cells. *Arterioscler Thromb Vasc Biol* **2017,** 37, (6), 1168-1179.
5. Marzioni, D.; Crescimanno, C.; Zaccheo, D.; Coppari, R.; Underhill, C. B.; Castellucci, M., Hyaluronate and CD44 expression patterns in the human placenta throughout pregnancy. *European journal of histochemistry : EJH* **2001,** 45, (2), 131-40.
6. Obut, M.; Oglak, S. C., Expression of CD44 and IL-10 in normotensive and preeclamptic placental tissue. *Ginekol Pol* **2020,** 91, (6), 334-341.
7. Takahashi, H.; Ogoyama, M.; Nagayama, S.; Suzuki, H.; Ohkuchi, A.; Matsubara, S.; Takizawa, T., Extravillous trophoblast invasion accelerated by WNT3A, 5A, and 10B via CD44. *J Matern Fetal Neonatal Med* **2021,** 34, (20), 3377-3385.
8. Todd, N.; McNally, R.; Alqudah, A.; Jerotic, D.; Suvakov, S.; Obradovic, D.; Hoch, D.; Hombrebueno, J. R.; Campos, G. L.; Watson, C. J.; Gojnic-Dugalic, M.; Simic, T. P.; Krasnodembskaya, A.; Desoye, G.; Eastwood, K. A.; Hunter, A. J.; Holmes, V. A.; McCance, D. R.; Young, I. S.; Grieve, D. J.; Kenny, L. C.; Garovic, V. D.; Robson, T.; McClements, L., Role of A Novel Angiogenesis FKBPL-CD44 Pathway in Preeclampsia Risk Stratification and Mesenchymal Stem Cell Treatment. *The Journal of clinical endocrinology and metabolism* **2021,** 106, (1), 26-41.
9. Shangguan, Y.; Wang, Y.; Shi, W.; Guo, R.; Zeng, Z.; Hu, W.; Cai, W.; Yan, Q.; Xu, Y.; Tang, D.; Dai, Y., Systematic proteomics analysis of lysine acetylation reveals critical features of placental proteins in pregnant women with preeclampsia. *J Cell Mol Med* **2021,** 25, (22), 10614-10626.
10. Muragaki, Y.; Abe, N.; Ninomiya, Y.; Olsen, B. R.; Ooshima, A., The human alpha 1(XV) collagen chain contains a large amino-terminal non-triple helical domain with a tandem repeat structure and homology to alpha 1(XVIII) collagen. *The Journal of biological chemistry* **1994,** 269, (6), 4042-6.
11. Nasu, K.; Fujisawa, K.; Nishida, Y.; Kai, S.; Sugano, T.; Miyakawa, I.; Tateishi, Y., Expression of collagen XVIII mRNA and protein in human umbilical vein and placenta. *Reproduction, fertility, and development* **2003,** 15, (1-2), 107-14.

12. Van Sinderen, M.; Cuman, C.; Winship, A.; Menkhorst, E.; Dimitriadis, E., The chrondroitin sulfate proteoglycan (CSPG4) regulates human trophoblast function. *Placenta* **2013,** 34, (10), 907-12.
13. Pulkkinen, L.; Alitalo, T.; Krusius, T.; Peltonen, L., Expression of decorin in human tissues and cell lines and defined chromosomal assignment of the gene locus (DCN). *Cytogenetics and cell genetics* **1992,** 60, (2), 107-11.
14. Siddiqui, M. F.; Nandi, P.; Girish, G. V.; Nygard, K.; Eastabrook, G.; de Vrijer, B.; Han, V. K.; Lala, P. K., Decorin over-expression by decidual cells in preeclampsia: a potential blood biomarker. *Am J Obstet Gynecol* **2016,** 215, (3), 361 e1-361 e15.
15. Daglar, K.; Kirbas, A.; Timur, H.; Ozturk Inal, Z.; Danisman, N., Placental levels of total oxidative and anti-oxidative status, ADAMTS-12 and decorin in early- and late-onset severe preeclampsia. *J Matern Fetal Neonatal Med* **2016,** 29, (24), 4059-64.
16. Uzun Cilingir, I.; Varol, F.; Gurkan, H.; Sutcu, H.; Atli, E.; Eker, D.; Inan, C.; Erzincan, S.; Sayin, C., Placental and serum levels of human Klotho in severe preeclampsia: A potential sensitive biomarker. *Placenta* **2019,** 85, 49-55.
17. Murthi, P.; van Zanten, D. E.; Eijsink, J. J.; Borg, A. J.; Stevenson, J. L.; Kalionis, B.; Chui, A. K.; Said, J. M.; Brennecke, S. P.; Erwich, J. J., Decorin expression is decreased in first trimester placental tissue from pregnancies with small for gestation age infants at birth. *Placenta* **2016,** 45, 58-62.
18. Crescimanno, C.; Marzioni, D.; Paradinas, F. J.; Schrurs, B.; Muhlhauser, J.; Todros, T.; Newlands, E.; David, G.; Castellucci, M., Expression pattern alterations of syndecans and glypican-1 in normal and pathological trophoblast. *The Journal of pathology* **1999,** 189, (4), 600-8.
19. Li, Q.; Han, Y.; Xu, P.; Yin, L.; Si, Y.; Zhang, C.; Meng, Y.; Feng, W.; Pan, Z.; Gao, Z.; Li, J.; Yang, W., Elevated microRNA-125b inhibits cytotrophoblast invasion and impairs endothelial cell function in preeclampsia. *Cell Death Discov* **2020,** 6, 35.
20. Gunatillake, T.; Chui, A.; Fitzpatrick, E.; Ignjatovic, V.; Monagle, P.; Whitelock, J.; Zanten, D.; Eijsink, J.; Borg, A.; Stevenson, J.; Brennecke, S. P.; Erwich, J.; Said, J. M.; Murthi, P., Decreased placental glypican expression is associated with human fetal growth restriction. *Placenta* **2019,** 76, 6-9.
21. Khan, S.; Blackburn, M.; Mao, D. L.; Huber, R.; Schlessinger, D.; Fant, M., Glypican-3 (GPC3) expression in human placenta: localization to the differentiated syncytiotrophoblast. *Histology and histopathology* **2001,** 16, (1), 71-8.
22. Deyssenroth, M. A.; Li, Q.; Lacasana, M.; Nomura, Y.; Marsit, C.; Chen, J., Expression of placental regulatory genes is associated with fetal growth. *J Perinat Med* **2017,** 45, (7), 887-893.
23. Rohde, L. H.; Janatpore, M. J.; McMaster, M. T.; Fisher, S.; Zhou, Y.; Lim, K. H.; French, M.; Hoke, D.; Julian, J.; Carson, D. D., Complementary expression of HIP, a cell-surface heparan sulfate binding protein, and perlecan at the human fetal-maternal interface. *Biology of reproduction* **1998,** 58, (4), 1075-83.
24. Grover, J.; Chen, X. N.; Korenberg, J. R.; Roughley, P. J., The human lumican gene. Organization, chromosomal location, and expression in articular cartilage. *The Journal of biological chemistry* **1995,** 270, (37), 21942-9.
25. Liu, C.; Hu, Y.; Wang, Z.; Pan, H.; Ren, Y.; Li, X.; Liu, Z.; Gao, H., The Downregulation of Placental Lumican Promotes the Progression of Preeclampsia. *Reprod Sci* **2021,** 28, (11), 3147-3154.
26. Li, N.; Hou, R.; Liu, C.; Yang, T.; Qiao, C.; Wei, J., Integration of transcriptome and proteome profiles in placenta accreta reveals trophoblast over-migration as the underlying pathogenesis. *Clin Proteomics* **2021,** 18, (1), 31.
27. Jokimaa, V.; Inki, P.; Kujari, H.; Hirvonen, O.; Ekholm, E.; Anttila, L., Expression of syndecan-1 in human placenta and decidua. *Placenta* **1998,** 19, (2-3), 157-63.
28. Szabo, S.; Xu, Y.; Romero, R.; Fule, T.; Karaszi, K.; Bhatti, G.; Varkonyi, T.; Varkonyi, I.; Krenacs, T.; Dong, Z.; Tarca, A. L.; Chaiworapongsa, T.; Hassan, S. S.; Papp, Z.; Kovalszky, I.; Than, N.

G., Changes of placental syndecan-1 expression in preeclampsia and HELLP syndrome. *Virchows Arch* **2013,** 463, (3), 445-58.

29. Garcha, D.; Walker, S. P.; MacDonald, T. M.; Hyett, J.; Jellins, J.; Myers, J.; Illanes, S. E.; Nien, J. K.; Schepeler, M.; Keenan, E.; Whigham, C. A.; Cannon, P.; Murray, E.; Nguyen, T. V.; Kandel, M.; Masci, J.; Murphy, C.; Cruickshank, T.; Pritchard, N.; Hannan, N. J.; Brownfoot, F.; Roddy Mitchell, A.; Middleton, A.; Pell, G.; Wong, G. P.; Tong, S.; Kaitu'u-Lino, T. J., Circulating syndecan-1 is reduced in pregnancies with poor fetal growth and its secretion regulated by matrix metalloproteinases and the mitochondria. *Sci Rep* **2021,** 11, (1), 16595.
30. Chui, A.; Zainuddin, N.; Rajaraman, G.; Murthi, P.; Brennecke, S. P.; Ignjatovic, V.; Monagle, P. T.; Said, J. M., Placental syndecan expression is altered in human idiopathic fetal growth restriction. *Am J Pathol* **2012,** 180, (2), 693-702.
31. Jeyarajah, M. J.; Jaju Bhattad, G.; Kops, B. F.; Renaud, S. J., Syndecan-4 regulates extravillous trophoblast migration by coordinating protein kinase C activation. *Sci Rep* **2019,** 9, (1), 10175.
32. Jones, R. L.; Salamonsen, L. A.; Zhao, Y. C.; Ethier, J. F.; Drummond, A. E.; Findlay, J. K., Expression of activin receptors, follistatin and betaglycan by human endometrial stromal cells; consistent with a role for activins during decidualization. *Molecular human reproduction* **2002,** 8, (4), 363-74.
33. Ciarmela, P.; Florio, P.; Toti, P.; Grasso, D.; Santopietro, R.; Tosi, P.; Petraglia, F., Expression of betaglycan in pregnant tIssues throughout gestation. *European journal of endocrinology* **2003,** 149, (5), 433-7.
34. Yi, Y.; Zhu, H.; Klausen, C.; Chang, H. M.; Inkster, A. M.; Terry, J.; Leung, P. C. K., Dysregulated BMP2 in the Placenta May Contribute to Early-Onset Preeclampsia by Regulating Human Trophoblast Expression of Extracellular Matrix and Adhesion Molecules. *Front Cell Dev Biol* **2021,** 9, 768669.
